# MRC2014: Extensions to the MRC format header for electron cryo-microscopy and tomography

**DOI:** 10.1016/j.jsb.2015.04.002

**Published:** 2015-11

**Authors:** Anchi Cheng, Richard Henderson, David Mastronarde, Steven J. Ludtke, Remco H.M. Schoenmakers, Judith Short, Roberto Marabini, Sargis Dallakyan, David Agard, Martyn Winn

**Affiliations:** a National Resource for Automated Molecular Microscopy, Electron Microscopy Group, New York Structural Biology Center, 89 Convent Ave, New York, NY 10027, USA; b MRC Laboratory of Molecular Biology, Francis Crick Avenue, Cambridge Biomedical Campus, Cambridge CB2 0QH, United Kingdom; c Department of Molecular, Cellular, and Developmental Biology, University of Colorado, Boulder, CO 80309-0347, USA; d Verna and Marrs McLean Department of Biochemistry and Molecular Biology, National Center for Macromolecular Imaging, Baylor College of Medicine, 1 Baylor Plaza, Houston, TX 70030, USA; e FEI, Achsteweg Noord 5, 5600 KK Eindhoven, The Netherlands; f Univ Autonoma Madrid, Escuela Politécnia Superior, E-28049 Madrid, Spain; g Howard Hughes Medical Institute and the Department of Biochemistry and Biophysics, University of California at San Francisco UCSF, MC 2240 600 16th Street, Room S412D, San Francisco, CA 94158-2517, USA; h Scientific Computing Department, STFC Daresbury Laboratory, Daresbury, Warrington WA4 4AD, United Kingdom

**Keywords:** 3DEM, three-dimensional electron microscopy, MX, macromolecular crystallography, Electron microscopy, Macromolecular crystallography, File format, Image data

## Abstract

The MRC binary file format is widely used in the three-dimensional electron microscopy field for storing image and volume data. Files contain a header which describes the kind of data held, together with other important metadata. In response to advances in electron microscopy techniques, a number of variants to the file format have emerged which contain useful additional data, but which limit interoperability between different software packages. Following extensive discussions, the authors, who represent leading software packages in the field, propose a set of extensions to the MRC format standard designed to accommodate these variants, while restoring interoperability. The MRC format is equivalent to the map format used in the CCP4 suite for macromolecular crystallography, and the proposal also maintains interoperability with crystallography software. This Technical Note describes the proposed extensions, and serves as a reference for the standard.

## 1 Introduction

Electron cryo-microscopy is a rapidly advancing technique in structural biology. Together with advances in sample preparation and in instrumentation, there are increasing efforts in software. The processing and interpretation of micrographs requires the serialization of a number of data objects into files for storage. These include the original and filtered micrographs, particle images, reconstructed single particle volumes, tomograms and sub-tomograms. Associated file formats need to store this data efficiently, and provide a clear description of the characteristics and provenance of the data.

The MRC file format for image and volume data describes an uncompressed single file with a defined header followed by a single data block. The format was created at the Medical Research Council Laboratory of Molecular Biology (MRC-LMB) in the 1980s to handle images and volumes obtained from 3DEM, and continues to be used in the suite of programs distributed by the MRC-LMB (Crowther et al., 1996). To maintain compatibility with the software library for electron density maps used by the Collaborative Computational Project No. 4 (CCP4) in MX (Winn et al., 2011), agreement between the two communities was reached in 1982 on a shared format specification. An update to this format was agreed in 2000 to include a machine stamp in the file header, to aid file portability.

The MRC format is widely used in the 3DEM field because of its simplicity and efficiency. It is used by the EMDataBank (Lawson et al., 2011) for the deposition and distribution of 3DEM volumes. As the 3DEM field has expanded, however, several variants of the MRC format have emerged. This divergence has come about as a result of the desire to record more metadata in the header, as well as to minimize the disk space taken up by the ever-increasing image sizes. Since these modifications were not coordinated, it has reached a point today that some of the variants, in particular those output from some image acquisition software packages, cannot be read by other packages.

The single data block of the MRC format naturally accommodates single volumes, single images or image stacks. The data is logically divided into sections, which may represent slices through a single volume or individual images of an image stack. In the era of sub-tomogram averaging and multiple 3D volume refinement, there is now a great desire to expand the dimension of the data stored to allow for a stack of volumes (so-called 4D data). The consensus has been that it would be impossible to represent volume stacks without a large change to the MRC format.

The authors have formed an e-mail discussion group since February 2013 to address these issues, and we present here our proposal for an update to the MRC standard. The changes affect not only all existing variants of the MRC format, but also the format used in CCP4 for MX. In coming up with these solutions, we have tried to minimize back compatibility issues within each of the MRC variants while improving uniform readability of the basic data and metadata across variants. The format update is being supported by changes to the MRC and CCP4 software libraries.

## 2 Overview of the MRC file format

The MRC format describes a binary file consisting of three parts. The first part, the main header, contains fixed format values for metadata about the images/volumes. The main header is limited to 1024 bytes, but includes unassigned space in anticipation of future extensions. The second part is a variable length extended header, originally designed to include symmetry operators for crystallographic applications, with the length of the extended header dependent on the relevant spacegroup. Finally, the third part contains the actual image/volume data, with grid values represented as one of a range of possible data types, according to the “mode” of the map.

The MRC standard is now maintained by the Collaborative Computational Project for Electron cryo-Microscopy (CCP-EM, Wood et al., 2007) and is available for reference at http://www.ccpem.ac.uk/mrc_format/mrc_format.php. This standard is based on the extension agreed in 2000, and is often referred to as the “new format”. Table 1 provides a short list of the variables relevant to this manuscript, together with their word and byte locations in the main header.

## 3 Current extensions to the MRC standard

### 3.1 Data modes

The original format specification listed modes 0–4 (word 4, see Table 1) as possible data types. Mode 0 represents a 1-byte integer. There has been some confusion as to whether this should be signed or unsigned, but the consensus is for signed. Modes 1 and 2 represent signed 2-byte integers and 4-byte reals, respectively. Modes 3 and 4 represent complex numbers, consisting of pairs of 2-byte integers and 4-byte reals, respectively. The CCP4 documentation lists a mode 5 but this is not currently used in CCP4, and to our knowledge has never been used.

Since the original specification, a number of new modes have been implemented by individual programs. Mode 6 for unsigned 2-byte integers has been implemented by UCSFtomo (Zheng et al., 2007), IMOD (Kremer et al., 1996) and EPU (FEI). For light microscopy, IVE (Image Visualization Environment, Chen et al., 1996) software stores images in the MRC-derived Imsubs format which uses Mode 7 for signed 4-byte integers. Finally, IMOD uses Mode 16 to represent RGB data in 3 unsigned bytes.

### 3.2 Extensions from microscope manufacturers

While microscope manufacturers typically have their own proprietary formats, many output an MRC-like format. The EPU package from FEI for automated Single Particle data acquisition outputs three files per image: a small version of the image in JPEG format, an XML file containing parameters of the data acquisition, and the image data itself as an “extended MRC” file. The Xplore3D package for electron tomography also generates an image stack in the same extended MRC format. These files currently include a 128 bytes extended header entry per image, located between the main header and the data block, which includes additional metadata such as tilt angles, stage position, exposure time, etc. The extended header has a fixed size of 1024 entries if NZ ⩽ 1024 and 8192 entries if NZ > 1024. The main header conforms to the original 1982 MRC/CCP4 specification, and is therefore incompatible with the current specification containing a machine stamp.

During initial development of Xplore3D in 2001, this MRC/CCP4 standard did not provide an unsigned 2-byte integer mode, so it was decided to first subtract 32768 to cast any unsigned data to signed before storage in mode 1. The inverse operation should be applied when reading this data back in memory. The distinction between image (stacks) and volumes is made based on file extensions: .rec for volumes, .mrc or .ali for images and (aligned) image stacks. For the more recently developed EPU software, the data may use the non-standard mode 6 for unsigned 2-byte integers. The first text label at the end of the main header is used to identify the file as coming from FEI software. Amira and Avizo are able to read and write these file types as well. EMAN2 (Tang et al., 2007) and IMOD also support reading of FEI MRC-like image files.

The Digital Micrograph software from Gatan outputs another variant of the MRC format. These files follow the IMOD convention (see below) including the use of an extended header for image stacks acquired in a tilt series.

### 3.3 Extensions for electron tomography

For input tomography tilt series and aligned images, IMOD (Kremer et al., 1996) supports two extended header sub-formats. In the UCSFtomo (Zheng et al., 2007) format, there is a series of 4-byte integers then real numbers for each image, with the number of each type per image listed in two 2-byte integers at Word 33. In practice, both UCSFtomo and Xplore3D use only real numbers, and IMOD expects to find several values in standard locations, particularly the tilt angle. In the SerialEM (Mastronarde, 2005) format, the extended header is a series of short integers for each image. These can consist of tilt angles, coordinates for montaged images, or a few other selected microscope parameters, with the number of bytes per image and bit flags for which items are included listed in two 2-byte integers in Word 33. SerialEM now stores much more extensive metadata in a text file with a simple key-value pair format that allows for both global data and data for each image.

Tomograms produced by IMOD (.rec files) do not have an extended header and do not use the non-standard Mode 6. Files written by IMOD since version 4.3 (2011) have the imodStamp (Word 39) set to indicate that the following field (Word 40) contains a set of defined bit flags. Bit 0 of the flags word is set to indicate that bytes are stored as signed numbers. IMOD will switch to writing bytes as signed in version 4.9 (2015), in line with the preferred definition of Mode 0; a temporary switch in 2014 revealed incompatibility with some existing software. Image stacks produced by SerialEM did not contain the imodStamp until recently (version 3.3, 2013), but this lack has little consequence, since camera images are rarely written as bytes.

## 4 Proposed extensions to the MRC/CCP4 standard

### 4.1 16-Bit unsigned integer data type

As discussed above, a 16-bit unsigned integer data type has long existed in files generated by electron tomography packages such as IMOD (Kremer et al., 1996) and UCSFTomo (Zheng et al., 2007), specified as MODE 6 in the header (word 4, see Table 1). The MRC/CCP4 standard does not define this datatype (MODE 1 defines a 16-bit *signed* integer data type). Nor does it give any other definition of MODE 6. We propose that the MRC/CCP4 standard is extended to include this *de facto* definition of MODE 6.

### 4.2 Extended header handling

Word 24 contains the item NSYMBT in the MRC/CCP4 format (see Table 1), which gives the number of bytes used for symmetry data inserted between the main header (1024 bytes in length) and the data block. Several 3DEM packages that output MRC format now place an extended header in the same location, for example to hold tilt angles in a tilt series image stack (SerialEM, Mastronarde (2005) and UCSFTomo, Zheng et al. (2007)), or to hold imaging conditions (EPU/Xplore3D). This usage is contrary to the current official MRC/CCP4 specification, which defines NSYMBT as being 0 or a multiple of 80, depending on the number of symmetry records held. In any case, without *a priori* knowledge of the particular extended header, it is impossible for 3rd party software to interpret or even preserve the extended header information.

We propose that NSYMBT is used to indicate the length of the extended header, *whether the extended header contains symmetry records or additional metadata, and with no constraint on the length*. Any software can use this value to skip the extended header and locate the data block. This mechanism is already used by the three packages mentioned above.

In addition, we propose a 4-character string EXTTYP at Word 27 which will be used to identify each extended metadata type (Table 2). Third party software can optionally use this information to interpret the contents of the extended header. In particular, CCP4 software will use this header entry to confirm that the extended header indeed contains symmetry records.

### 4.3 Volume stack definition

**Fig. 1:**
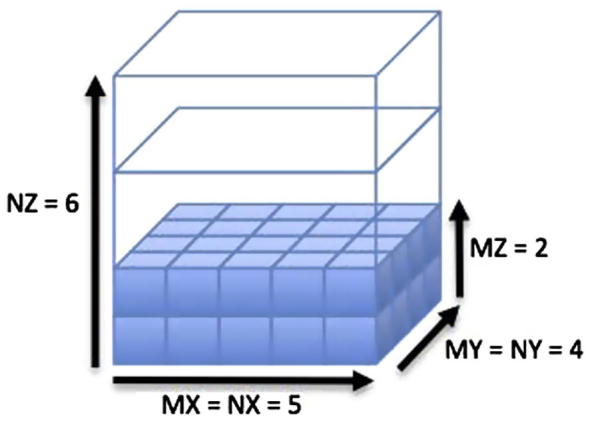
An example of the volume stack specified by NX/NY/NZ and MX/MY/MZ. In this simple example, there are 3 volumes, each consisting of 2 sections.

To date, images and volumes have been distinguished solely by the ISPG variable at Word 23 (Table 1), with images and image stacks having ISPG 0 while single EM volumes have ISPG 1. As such, it is not possible to explicitly specify a volume stack. We have agreed to use two devices (Table 3) to solve this problem:

I. Use spacegroup + 400 to represent a 4D volume stack. Typically a stack of EM volumes will be assigned ISPG 401. In principle, if a file contains a stack of volumes each possessing (the same) crystal symmetry, then 401 ⩽ ISPG ⩽ 630 would be used.
II. The MZ variable at Word 10 represents the number of intervals, or sampling grid, along Z in a crystallographic unit cell in CCP4, typically used in maps created by Fourier synthesis. A particular map held in a CCP4-format file may have more (NZ > MZ) or fewer (NZ < MZ) sections than this. Where crystallography does not apply, software in 3DEM field has typically ignored this field or simply assumed that it is the same as NZ (total number of sections). We propose to use MZ to identify the number of sections along Z in each of the single volumes that makes up a volume stack (Fig. 1). By adopting this unit-cell-like definition, we have
  (1) NZ=MZ∗number of volumes

where NZ is the total number of sections in Z found in Word 3.

Using these two devices, different types of data can be told apart as shown in Table 3.

### 4.4 Voxel size

Following the new (or restored) definition of MZ, we need to clarify the definition of voxel size. Since NZ = MZ in the case of single volume, the voxel size in Z could be derived from the ratio of the third CELLA value (Word 13) to either integer, as in the case of the *X* and *Y* axes. In the case of a volume stack, the situation is potentially unclear. For future consistency, we propose that the voxel size in each dimension is defined by the corresponding ratio of the length in CELLA (words 11–13) in Angstroms to the number of intervals MX/MY/MZ (words 8–10). Thus,

(2) Voxel size in Z in Angstroms=3rdCELLA/MZ

This definition is already used in CCP4 and IMOD, as well as in popular 3D rendering programs such as UCSF Chimera (Pettersen et al., 2004). In addition, it reduces the header changes upon addition of a new volume to the volume stack to NZ alone.

### 4.5 Indication of undetermined density statistics

Statistical parameters of the density values in the image/map are recorded in the header in Word 20 (DMIN), 21 (DMAX), 22 (DMEAN), and 55 (RMS). While these values are expected to be accurate for a single image or volume, they are often undetermined or inaccurately determined for large image and volume stacks since tracking of the changes in these statistics is time-consuming, especially when members of the stack are deleted or inserted. We have agreed that undetermined statistics for stacks will be indicated in the following manner:

1. DMAX < DMIN indicates that minimum and maximum of density are not determined.
2. DMEAN < (smaller of DMIN and DMAX) indicates that mean value of the density is not determined.
3. RMS < 0 indicates that RMS deviation of map from mean is not determined.

While most packages we are involved in assume that the density statistics are unreliable and re-evaluate them upon reading for critical calculations, we recommend that minimal stack statistics of inclusive DMIN and DMAX are always recorded in the file for the purpose of scaling map display in case an accurate determination is not feasible.

### 4.6 Clarification of the definition of ORIGIN field

We agreed to clarify the definition of the ORIGIN field. For transforms (Mode 3 or 4), this field is the phase origin of the transformed image in pixels, e.g. as used in helical processing of the MRC package. For a transform of a padded image, this value corresponds to the pixel position in the padded image of the center of the unpadded image. For real-space images (Mode 0, 1, 2 and 6), this field has been used by some packages in tomography to define the origin of a coordinate system that is invariant after scaling, padding, or cropping images. However, IMOD and Chimera differ in their sign convention in this context. We recommend Chimera convention in future development. Chimera defines the field as the position of the outside corner of the first pixel of the file relative to the origin of the coordinate system, expressed in real-space units (Angstroms) rather than pixels. A cropped image is given a positive origin value to indicate that it is taken from a coordinate inside the original image. Such sign convention is the same as the sign convention of the integer START fields (Words 5–7). IMOD currently uses the opposite sign convention but has already defined a bit in its flags field (bit 2 of Word 40) to indicate whether the origin follows the new convention. This flag will allow it to switch to the new convention by 2017.

### 4.7 Version indication

To assist future changes to the format, we dedicate Word 28 (NVERSION, Int32) to identifying the version of the MRC format that the file adheres to, specified as:

Year∗10+version within the year(base0)

For the current format change, the value would be 20140.

Software that writes out an MRC file can add the number in this field to indicate its compliance to the format standard and to provide other programs a quick check point. Older or unverified files will have the default value of 0 in this field.

## 5 Implementation

We desire that the MRC/CCP4 format is consistent across 3DEM and MX, so that volumes can be passed seamlessly between software packages in both fields. The changes proposed here must therefore be implemented in the CCP4 software library, as well as in 3DEM software.

The current CCP4 library maps values of the MODE variable (word 4) to low-level I/O routines. There is currently no I/O support for a MODE 6 file, and this will be added. The CCP4 library already uses NSYMBT to locate the data block, but support for the new EXTTYP word needs to be added. If the EXTTYP is set, and has a value different from ‘CCP4’ or ‘MRCO’, then the CCP4 library will not attempt to interpret the extended header in terms of symmetry records. Thus, in time the CCP4 library will be able to read files from more 3DEM packages, but the priorities are set conservatively so as not to break crystallographic usage. In this context, it should be noted that in crystallography maps are increasingly calculated on the fly from Fourier coefficients, rather than stored in files.

CCP4 defines the standard spacegroups 1–230, and a number of common alternative settings. The latter are indicated by adding a multiple of 1000 to the standard spacegroup numbers, e.g. alternative settings of spacegroup 17 are defined as 1017 and 2017. There is thus no conflict in principle in using numbers 401–630 for volume stacks. The CCP4 library has now been changed to recognize these spacegroup numbers, and to identify the kind of data according to the specifications of Table 3.

3DEM packages not using the CCP4 library to read images and volumes will also need modification. For example, in Appion (Lander et al., 2009) where NSYMBT is already used to locate the data block, the voxel definition needs to be adjusted to that given in Eq. (2), and segmenting volume stacks according to Eq. (1) needs to be added. From version 3.1, XMIPP (de la Rosa-Trevin et al., 2013) has adopted the conventions shown in Table 3. As of version 4.7.7, IMOD follows the conventions proposed here. The Tilt program now sets ISPG to 1 when generating a tomogram. The Alterheader program can be used to modify ISPG and MZ appropriately for a volume stack, and the Newstack program will retain this information when copying volumes. As of version 3.4.4, SerialEM places ‘SERI’ in the proposed EXTTYPE field. FEI will adopt mode 6 in future releases of Xplore3D to avoid the signed/unsigned conversion issues mentioned above, and will add the proposed conventions of Table 1, Table 2, Table 3 in future EPU, Xplore3D, Amira and Avizo releases.

Existing single images and single volumes generated without the extended header and following the MRC2000 definition of CELLA should be compatible with the proposed definitions with no change required. For those with an extended header, addition of EXTTYP will be necessary in order to avoid confusion.

The case for image stacks is complicated by the fact that, in several packages, MZ is assigned to be the same as NZ. To maintain this back compatibility, we recommend that new developments use ISPG = 0 and NZ > 1 as an indication of image stack, rather than relying on MZ = 1.

## 6 Concluding remarks

The authors intend to adopt these extensions to the MRC/CCP4 format, and make the required changes to the following packages: CCP4, MRC library, Xplore3D, EPU, Amira/Avizo, IMOD, UCSFTomo, EMAN2, Appion and XMIPP. The revised format will be referred to as MRC2014, and curated and disseminated by the CCP-EM project at http://www.ccpem.ac.uk/mrc_format/mrc_format.php. We have also created a web server for developers to validate the modification involved in this proposal at http://emportal.nysbc.org/mrc2014/.

With the proposed revision of the MRC/CCP4 format regarding different extended headers and volume stack definition, we hope to achieve basic volume/image data exchangeability. We did not attempt to reach agreement on the type and the format of metadata stored in an extended header: that issue remains in the control of each individual variant, and we caution that metadata stored in these extended header may be lost unless the reader program is made to read the particular variant. Also note that we did not discuss some other fields in the header whose usage and interpretation varies among software packages. If consensus is reached at a later date, then a new version of the format will be released and advertised widely in the community. This process will be managed by CCP-EM, and the procedure is detailed on the website at http://www.ccpem.ac.uk/mrc_format/mrc_format.php.

Finally, we note that there are a large number of file extensions in use for MRC format files, which should not be necessary for identifying the contents of a file. We suggest that the extensions .mrc and .map should be preferred, but acknowledge that a particular naming convention may be convenient in the context of a particular software suite.

The MRC/CCP4 format is widely used, and a strong specification of the format will ensure that its use is not problematic. Nevertheless, we recognize that the limited extensibility of the header and the single data block mean that it is not as flexible as one would wish these days. There may be good reasons for the community to adopt a more modern format for 3DEM data objects. In the meantime, we hope that these proposals will ease the problems of practising scientists.

## Figures and Tables

**Table 1:** Selected entries in the main header of the MRC format relevant to the proposal described here. The word and byte position in the main header are given in the first two columns (numbered from 1). The third column gives the data type, and the fourth gives the usual name of the entry, although only the value appears in the actual file header. The final column gives a short description of the entry.

Long word #	Byte #	Data type	Name	Description
1	1–4	Int32	NX	Number of columns
2	4–8	Int32	NY	Number of rows
3	9–12	Int32	NZ	Number of sections
4	13–16	Int32	MODE	Data type
…				
8	29–32	Int32	MX	Number of intervals along *X* of the “unit cell”
9	33–36	Int32	MY	Number of intervals along *Y* of the “unit cell”
10	37–40	Int32	MZ	Number of intervals along *Z* of the “unit cell”
11–13	41–52	Float32	CELLA	Cell dimension in angstroms
…				
20	77–80	Float32	DMIN	Minimum density value
21	81–84	Float32	DMAX	Maximum density value
22	85–88	Float32	DMEAN	Mean density value
23	89–92	Int32	ISPG	Space group number 0, 1, or 401
24	93–96	Int32	NSYMBT	Number of bytes in extended header
…				
27	105–108	Char	EXTTYPE	Extended header type
28	109–112	Int32	NVERSION	Format version identification number
…				
50–52	197–208	Float32	ORIGIN	Origin in *X*, *Y*, *Z* used in transform
53	209–212	Char	MAP	Character string ‘MAP’ to identify file type
54	213–216	Char	MACHST	Machine stamp
55	217–220	Float32	RMS	RMS deviation of map from mean density
…				

**Table 2:** Initial EXTTYPE assignments for extended metadata generated by different software, specified at word 27. This entry specifies the contents of the extended header, while NSYMBT (word 24) specifies the length. The list of values can be extended in future.

EXTTYP	Format origin
CCP4	CCP4
MRCO	Original MRC
SERI	SerialEM
AGAR	Agard
FEI1	FEI software, e.g. EPU and Xplore3D, Amira, Avizo

**Table 3:** Summary of the MRC format specification for 3DEM data of different dimensions. ISPG is the spacegroup number given at word 23, MZ is the sampling along the *z* axis given at word 10, and NZ is the number of sections along the *z* axis given at word 3.

Data dimension type	ISPG	MZ
Single image	0	1
Image stack	0	⩾1
Single volume	1	=NZ
Volume stack	401	=NZ/number of single volumes
